# Batch Blast Extractor: an automated blastx parser application

**DOI:** 10.1186/1471-2164-9-S2-S10

**Published:** 2008-09-16

**Authors:** Mehdi Pirooznia, Edward J Perkins, Youping Deng

**Affiliations:** 1 Department of Biological Sciences, University of Southern MS, Hattiesburg, 39406, USA; 2 Environmental Laboratory, U.S. Army Engineer Research and Development Center, 3909 Halls Ferry Rd, Vicksburg, MS, 39180, USA

## Abstract

**Motivation:**

BLAST programs are very efficient in finding similarities for sequences. However for large datasets such as ESTs, manual extraction of the information from the batch BLAST output is needed. This can be time consuming, insufficient, and inaccurate. Therefore implementation of a parser application would be extremely useful in extracting information from BLAST outputs.

**Results:**

We have developed a java application, Batch Blast Extractor, with a user friendly graphical interface to extract information from BLAST output. The application generates a tab delimited text file that can be easily imported into any statistical package such as Excel or SPSS for further analysis. For each BLAST hit, the program obtains and saves the essential features from the BLAST output file that would allow further analysis. The program was written in Java and therefore is OS independent. It works on both Windows and Linux OS with java 1.4 and higher. It is freely available from:

## Background

The NCBI BLAST database search tool is one of the most popular programs designed to solve single query problems. BLAST (Basic Local Alignment Search Tool) is the heuristic search algorithm employed by the programs blastp, blastn, blastx, tblastn, and tblastx. The BLAST programs were tailored for sequence similarity searching for example to identify homologs of a given query sequence [1].

The five common BLAST programs perform the following tasks: 1) blastp compares an amino acid query sequence against a protein sequence database; 2) blastn compares a nucleotide query sequence against a nucleotide sequence database; 3) blastx compares the six-frame conceptual translation products of a nucleotide query sequence (both strands) against a protein sequence database 4) tblastn compares a protein query sequence against a nucleotide sequence database dynamically translated in all six reading frames (both strands), and 5) tblastx compares the six-frame translations of a nucleotide query sequence against the six-frame translations of a nucleotide sequence database.

**Figure 1:**
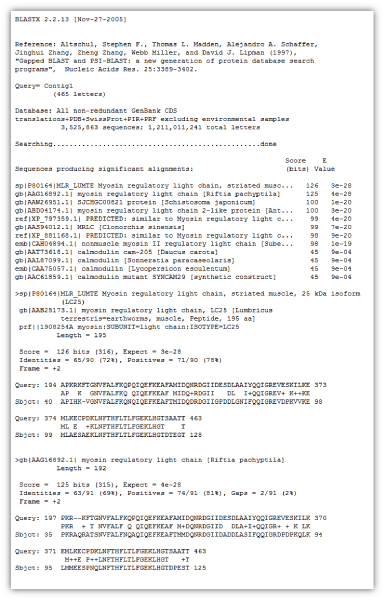
Screenshot of a Blastx Output.

The BLAST programs all provide information in roughly the same format. First comes (A) an introduction to the program; (B) a histogram of expectations if one was requested; (C) a series of one-line descriptions of matching database sequences; (D) the actual sequence alignments, and finally the parameters and other statistics gathered during the search. However, for genome-wide comparisons involving multiple queries (batch query), the search is a challenge. For instance, EST collections are currently produced for many species as an efficient strategy for gene identification. Analysis of the ESTs involves clustering, contig formation and annotation of thousands of fragments, interpretation of which may involve thousands of individual BLAST searches [2-5]. An automated post processing of the output (Figure 1) can simplify the analysis in such cases. The blast parser (BlastLikeSaxParser) in BioJava [6] and BPlite from BioPerl [7] are frequently being used to parse a variety of different blast outputs, but neither are user friendly and therefore programming skills are needed to use these applications.

**Figure 2:**
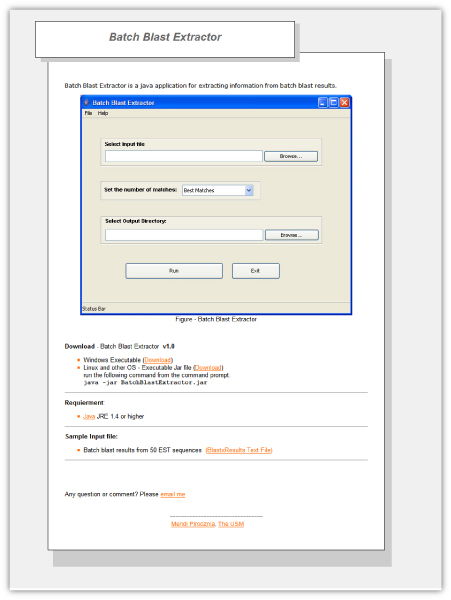
Screenshot of the Batch Blast Extractor Web site.

**Figure 3:**
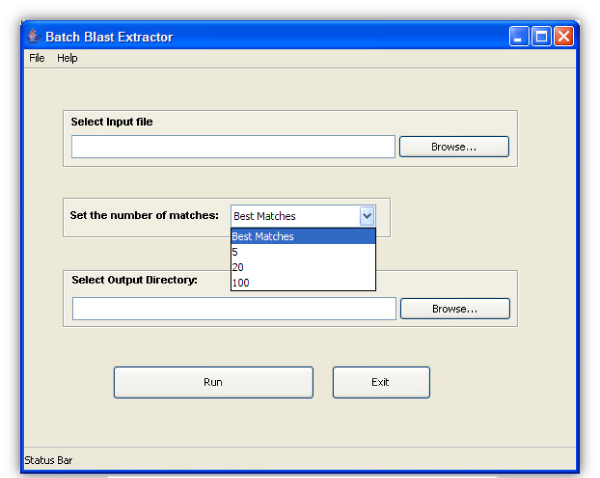
The Bach Blast Extractor Graphical User Interface.

We developed the "Batch Blast Extractor" program (Figure 2 and 3) for use in this regard. It serves as a parser storing only the essential features of BLAST hits in a tabular form. The user can then apply a number of selection criteria to filter out hits with particular attributes. "Batch Blast Extractor" thus serves as a powerful annotation tool for large sets of query sequences.

## Results

The application generates a tab delimited text file that can be easily imported into any statistical package such as Excel or SPSS for further analysis. For each BLAST hit, the program derives and saves the following features: Query ID, Query Length, Accession version and GI number, Alignment Length, Score, bit, E-value, Identities, Positives, Gaps, Frame, Organism, and Description.

The extracted information includes the following:

▪ Query: headers of sequences to analyze

▪ Subject: headers of sequences found in the database

▪ Score: a number representation (e.g. 550)

▪ Score Text: full text representation plus BITS (e.g. 235 bits (450))

▪ Expect: the E-Value as number (e.g. 1e-166)

▪ Identities %: a number representation (e.g. 85)

▪ Identities Text: full text representation plus characters matching (e.g. 110/130 (90%))

▪ Positives %: a number representation (e.g. 92)

▪ Positives Text: full text representation (e.g. 110/130 (90%))

▪ Gaps %: a number representation (e.g. 11)

▪ Gaps Text: full text representation plus voids (e.g. 9/102 (9%))

▪ Frame: orientation of the translated ORF (e.g. +3)

▪ Length Query: the number of nucleotides or amino acids (e.g. 400)

▪ Length Subject: the number of nucleotides or amino acids (e.g. 500)

▪ Position Query: as text representation plus the length of the frame (e.g. 328–600 (360))

▪ Position Subject: as text representation plus the length of the frame (e.g. 1–110 (120))

The program was written in Java. It is OS independent and works on both Windows and Linux OS with java 1.4 and higher. It is freely available to noncommercial users from:  (Figure 2 and 3).

Currently the application works with blastx results. Efforts to extend functionality to other BLAST programs such as blastp and blastn are in progress.

## Competing interests

The authors declare that they have no competing interests.

## Authors' contributions

MP and YD initiated the project. MP designed, programmed and implemented the application and drafted the manuscript. EJP and YP directed the project. All authors read and approved the final manuscript.
